# DEGAS: *De Novo* Discovery of Dysregulated Pathways in Human Diseases

**DOI:** 10.1371/journal.pone.0013367

**Published:** 2010-10-19

**Authors:** Igor Ulitsky, Akshay Krishnamurthy, Richard M. Karp, Ron Shamir

**Affiliations:** 1 Blavatnik School of Computer Science, Tel Aviv University, Tel Aviv, Israel; 2 University of California, Berkeley, California, United States of America; 3 International Computer Science Institute, Berkeley, California, United States of America; King Abdullah University of Science and Technology, Saudi Arabia

## Abstract

**Background:**

Molecular studies of the human disease transcriptome typically involve a search for genes whose expression is significantly dysregulated in sick individuals compared to healthy controls. Recent studies have found that only a small number of the genes in human disease-related pathways show consistent dysregulation in sick individuals. However, those studies found that some pathway genes are affected in most sick individuals, but genes can differ among individuals. While a pathway is usually defined as a set of genes known to share a specific function, pathway boundaries are frequently difficult to assign, and methods that rely on such definition cannot discover novel pathways. Protein interaction networks can potentially be used to overcome these problems.

**Methodology/Principal Findings:**

We present DEGAS (DysrEgulated Gene set Analysis via Subnetworks), a method for identifying connected gene subnetworks significantly enriched for genes that are dysregulated in specimens of a disease. We applied DEGAS to seven human diseases and obtained statistically significant results that appear to home in on compact pathways enriched with hallmarks of the diseases. In Parkinson's disease, we provide novel evidence for involvement of mRNA splicing, cell proliferation, and the 14-3-3 complex in the disease progression. DEGAS is available as part of the MATISSE software package (http://acgt.cs.tau.ac.il/matisse).

**Conclusions/Significance:**

The subnetworks identified by DEGAS can provide a signature of the disease potentially useful for diagnosis, pinpoint possible pathways affected by the disease, and suggest targets for drug intervention.

## Introduction

Systems biology has the potential to revolutionize the diagnosis and treatment of complex disease by offering a comprehensive view of the molecular mechanisms underlying their pathology. To achieve these goals, biologists need computational methods that extract mechanistic understanding from the masses of available data. To date, the main sources of such data are microarray measurements of genome-wide expression profiles, with over 400,000 profiles stored in GEO [1] alone as of April 2010. A wide variety of approaches for elucidating molecular mechanisms from expression data have been suggested [2], [3]. However, most of these methods are effective only when using expression profiles obtained under diverse conditions and perturbations, while the bulk of data currently available from clinical studies are expression profiles of groups of diseased individuals and matched controls. These data are useful for characterizing the molecular signature of a disease for diagnostic and prognostic purposes [4], [5]. However, using these expression profiles to obtain a better understanding for the pathogenesis is significantly more difficult. The standard methods applied to these data identify the genes that best predict the pathological status of the samples. While these methods are successful in identifying potent signatures for classification purposes, the mechanistic insights that can be obtained from examining the gene lists they produce are frequently limited [6].

Standard statistical tests, as well as the vast majority of more sophisticated methods utilizing diverse genomic data, look for genes whose expression is significantly and robustly different in the case and in the control cohorts. Several recent comprehensive studies, mostly in the context of cancer, have found that few genes meet these criteria. Yet, many of the affected individuals were found to carry dysregulated genes that belong to specific disease-related pathways [7], [8], [9], [10]. In order to identify such pathways, these studies utilized a fixed collection of gene lists based on current biological knowledge. While several computational methods have been developed for quantifying the changes in the expression levels of a gene set [11], [12], [13], [14], [15], [16], [17], [18], our knowledge of the true pathways is very incomplete, and pathway boundaries are often difficult to assign. In addition, frequently, only part of the pathway is altered during disease. Therefore, it is more desirable to be able to identify disease-related pathways *de novo*, without assuming prior knowledge of the pathways. The use of gene networks for finding disease-related pathways that form connected subnetwork has been suggested for this problem [19], [20]. The drawback of this approach is that it can only use genes that are connected to other pathway members through physical interactions. However, the appeal of using network information increases as the quality and scale of experimental data on such interaction networks improve [21].

Several approaches for integrating microarray measurements with network knowledge were described in the literature. Some (including us) proposed computational methods for detection of subnetworks that show correlated expression [22], [23], [24], [25]. A successful method for detection of ‘active subnetworks’ was proposed by Ideker et al. and extended by other groups [26], [27], [28], [29], [30]. These methods are based on assigning a significance score to every gene in every sample and looking for subnetworks with statistically significant combined scores. Breitling et al. proposed a simple method named GiGA which receives a list of genes ordered by their differential expression significance and extracts subnetworks corresponding to the most differentially expressed genes [31]. Other methods used differential expression scores assigned to individual genes and look for subnetworks with high aggregate scores [32], [33]. Other tools used network and expression information together for classification purposes [19], [20]. Finally, others used networks to identify novel disease-related genes based on their proximity to known disease related genes [34], [35], [36], [37], [38].

Methods based on correlated expression patterns do not use the sample labels, and thus their applicability for case-control data is limited, as correlation between transcript levels can stem from numerous confounding factors not directly related to the disease (e.g., age or gender). The extant methods that do use the sample labels rely on the assumption that the same genes in the pathway are differentially expressed in all the samples (an exception is jActiveModules, which can identify a subset of the samples in which the subnetwork is active [26]). This assumption may hold in simple organisms (e.g., yeast or bacteria) or in cell line studies. However, in human disease studies, the samples are expected to exhibit intrinsic differences due to genetic background, environmental effects, tissue heterogeneity, disease grade and other confounding factors. Thus, improved methods that can account for this variability and recover focused disease-affected pathways are needed.

Here we describe DEGAS (DysrEgulated Gene set Analysis via Subnetworks), a new method for analysis of clinical gene expression samples in the context of interaction networks, which avoids the above assumption. Given a set of expression profiles labeled as cases and another set of controls, DEGAS aims to detect subnetworks in which multiple genes are dysregulated in the cases, while allowing for distinct affected gene sets in each case profile. We call such modules *dysregulated pathways* (DPs). Specifically, for each gene, we use the distribution of values in the controls in order to determine in which cases that gene is dysregulated. We then look for minimal connected subnetworks of the given protein interaction network in which the number of dysregulated genes in each case exceeds a given threshold. By comparing to statistics of randomized networks, we can select a meaningful value for this threshold and identify statistically significant DPs. As finding DPs is computationally hard, we propose heuristics and algorithms with provable approximation ratios and study their performance. Our approach has several important advantages over the existing methods: (a) the dysregulated genes in a DP can vary between patients; (b) the method is robust to outliers (i.e., patients with unusual profiles); (c) the DPs can contain relevant genes based on their interaction pattern, even if they are not dysregulated; (d) it has a limited number of parameters, all of which have an intuitive biological interpretation; (e) while not guaranteeing optimality, the algorithmic core of the method has a provable performance guarantee.

In order to test the performance of our method, we collected 13 case-control gene expression datasets for seven diseases, and tested the ability of DEGAS and other methods [26], [31] to recover a pathway corresponding to the relevant disease pathway in the KEGG database [39]. Comparing our method to existing alternatives [26], [31] we find that DEGAS can identify more specific and focused subnetworks which capture a significant fraction of the known disease-related pathways. Using a dataset of gene expression in tongue squamous cell carcinoma we show how DEGAS can identify the known hallmarks of a well studied disease. We then focus on Parkinson's Disease (PD), which is relatively poorly understood on the molecular level, and show how DEGAS can suggest mechanisms that are affected in PD brain, some of which have support in other existing data. We obtain consistent results in two different PD datasets. Mainly, our results point to a previously unrecognized pathway-level dysregulation of mRNA splicing in PD patients.

A preliminary version of this paper appeared in [40]. This version differs in the exact problem formulation, the algorithmic details, and in the implementation. Moreover, the data analysis has been completely revised and this version contributes novel biological insights derived from DEGAS.

An implementation of DEGAS with a full graphical user interface for parameter specification and network visualization is available as part of the MATISSE network analysis software at http://acgt.cs.tau.ac.il/matisse.

## Results

### A framework for detection of pathways dysregulated in human disease

**Figure 1:**
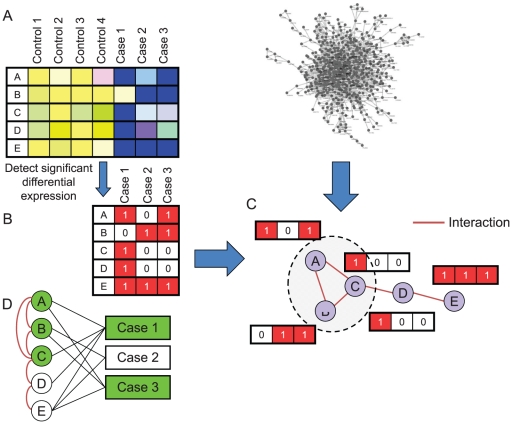
A dysregulated pathway (DP). (**A**) The input to our method consists of expression data of case and control cohorts and a protein interaction network. (**B**) The expression data are converted into a binary genes over cases matrix in which “1” appears in position (*i*,*j*) if gene *i* is dysregulated in case *j* (relative to the expression levels of *i* in the control cohort). (**C**) The interaction network: The vector next to each protein is the dysregulation status (0 or 1) of that gene in each case. A DP is a minimal subnetwork in which at least *k* genes are dysregulated in all but *l* cases. In the shown example, *k = 2* and *l* = 1. In the circled subnetwork, two out of the three genes are dysregulated in the first and the third case (the second case is the outlier). (**D**) An alternative representation of the data in C, as a bipartite graph. Genes are on the left and cases are on the right. The blue edges are protein interactions and the gray edges connect the genes with cases in which they are dysregulated.

In this section we describe the theoretical foundations of our methodology (**Figure 1**), which are detailed in the Methods section and in the **Text S1**. The input to our method consists of a network, which is an undirected and unweighted graph, and a collection of gene expression profiles, divided into ‘control’ and ‘case’ cohorts (**Figure 1A**). Each expression profile consists of the expression levels of some of the nodes in the network in one individual (some genes may not have expression data, e.g., because they were absent from the microarray). Our basic formulation defines a dysregulated pathway as the smallest connected subgraph in the network in which a specific number of genes are dysregulated for each case when compared to controls. We look for the smallest possible network, as it corresponds to the most focused ‘explanation’ of the disease in terms of gene expression. In other words, we are seeking clusters of gene expression dysregulation events in the network. See Methods for formal definitions.

In the first step, we identify, in each case profile, the set of genes that are dysregulated when compared to controls (**Figure 1B**, see Methods). Our goal is then to identify the smallest subgraph that contains (*covers*) at least *k* genes from each of those sets, except for up to *l* outlier sets, from which fewer genes can be present (**Figure 1C**). Our method thus has two main parameters *k* - the number of genes affected in the pathway in each individual, and *l* - the number of allowed outliers (cases excluded from the analysis).

**Figure 2:**
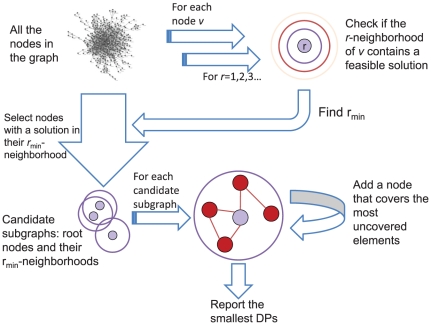
DEGAS outline. All the nodes in the network are tested as potential root nodes for a minimal radius DP. For each node, we efficiently compute the smallest radius for which some DP exists in the r-neighborhood of the node. All the nodes for which this radius is minimal are used to construct DP using the ExpandingGreedy heuristic (see Methods). The smallest DPs identified over all the tested roots are reported.

Our initial results have shown that, in this basic formulation, small DPs frequently correspond to sparse subnetworks that were frequently not biologically relevant (results not shown). We adjusted our problem formulation accordingly, and focused on identifying DPs that are not only small, but also have the smallest possible radius – i.e., all the nodes in a DP are within a short distance from some *root* node (**Figure 2**, see Methods).

### The DEGAS algorithm

The problem of identifying dysregulated pathways is related to the *set cover* problem, a classical problem known to be computationally hard. We have developed and compared three different algorithms for solving this problem. Two of these algorithms can identify pathways that are within a certain margin of error from the optimal solution, while the third can deliver arbitrarily large pathways in some specific problem instances. However, we found that, on biological data, the third algorithm augmented with a few heuristics tends to identify DPs that are significantly smaller (and therefore more biologically plausible) than the former two algorithms (see Methods for details). We therefore used this algorithm (which we refer to as DEGAS) in the rest of this paper.

DEGAS is described in detail in the Methods section. Abstractly, DEGAS consists of two phases. First, we identify nodes that could be potentially good starting points for the algorithm – these are nodes in the vicinity of which a possible solution can be found. The *r-* neighborhood of node *v* is the set of all the nodes that can be reached from *v* by a path that contains ≤*r* edges. We test all the nodes in the graph and find *r_min_* – the smallest *r* for which some node contains a proper DP in its *r* –neighborhood. Only those nodes for which a proper DP is found in their *r*_min_-neighborhood are considered as starting points in the second phase, in which for each such *root* node we perform a greedy search that attempts to find the smallest DP in the *r*_min_-neighborhood. Each search starts with a partial DP that contains only the root and iteratively expands it. For a partial DP, call the cases for which less than *k* genes are in the DP the *uncovered cases.* In every iteration, DEGAS searches the nodes neighboring the DP for a node that covers the largest number of uncovered cases and adds this node to the DP. The greedy search stops when the number of uncovered cases is at most *l*. The smallest DP(s) identified over all the searches are then returned.

Since the outliers are not specified in advance, the search algorithm may add to the DP surplus nodes that are covering cases which are eventually discarded. We attempt to deal with this problem by running the search algorithm twice – the set of outliers identified in the first round is ignored in the second execution, in which no further outliers are allowed (see Methods for details).

### Assessment of DP significance

One of the main issues determining the performance of DEGAS is the setting of the parameters. The *l* parameter (the number of outlier cases) can be set based on the *a priori* assessment of the homogeneity of the case cohort in the study. In all the analyses described here, we set *l* to 20% of the cases in the dataset. The setting of the *k* parameter (the number of genes affected in each case) is more difficult, since in the vast majority of human diseases, the number of critical dysregulation events in the affected pathway is unknown. Recall that our goal is to identify significant concentration of dysregulation events in a single pathway. We therefore decided that the best value for the k parameter will be the one for which the size of the smallest DP found is significantly smaller than that obtained in random networks. For each tested *k* value we computed the sizes of the DP found in the network, and used the distribution of the sizes of the DPs found in randomly permuted networks, to assign an empirical p-value, which reflects the fraction of random networks in which an equal-sized or smaller DP can be found. The parameter *k* yielding the most significant p-value is then reported.

### A compendium of disease pathways

A rigorous assessment of the performance of DEGAS required gene expression datasets consisting of cases and controls for a specific disease, as well as sets of genes known to be related to the disease. To this end, we assembled a collection of 13 datasets consisting of case and control gene expression profiles, for which a corresponding pathway was present in the KEGG database as of July 2009 [41], [42], [43], [44], [45], [46], [47], [48], [49] (**Table 1**). We used only datasets in which a healthy tissue was compared to the disease tissue (i.e., datasets with multiple prognosis-based cohorts were excluded). For uniformity, we only used datasets that employed the relatively widely used Affymetrix microarrays from the HG-U133 series. For each dataset, we used DEGAS to identify DPs up-regulated in cases compared to controls (“UP”), down-regulated in cases compared to controls (“DOWN”) or differentially expressed between the two cohorts (“DIFF”).

**Table 1:** Gene expression datasets used in this study.

Dataset	KEGG pathway	Reference	GEO accession	Number of cases	Number of controls
*AD*	Alzheimer's disease (AD)	[41]	GSE5281	10	13
*ASTHMA*	Asthma	[46]	GSE4302	42	28
*PYLORI*	Epithelial cell signaling in Helicobacter pylori infection	-	GSE5081	8	8
*HD*	Huntington's disease (HD)	[48]	GSE3790	38	32
*SUN-GLIOBLASTOMA*	Pathways in cancer	[47]	GSE4290	77	23
*SUN-ASTROCYTOMA*	Pathways in cancer	[47]	GSE4290	26	23
*SUN-OLIGODENDROGLIOMA*	Pathways in cancer	[47]	GSE4290	50	23
*ESTILO-OTSCC*	Pathways in cancer	[44]	GSE13601	31	26
*YE-OTSCC*	Pathways in cancer	[45]	GSE9844	26	12
*MORAN-PD*	Parkinson's disease (PD)	[42]	GSE8397	29	18
*LESNICK-PD*	Parkinson's disease (PD)	[43]	GSE7621	16	9
*SLE*	Systemic lupus erythematosus (SLE)	[49]	GSE8650	38	21

Each dataset contained a comparison of sick individuals and healthy controls. All the data were obtained from Gene Expression Omnibus (GEO, http://www.ncbi.nlm.nih.gov/geo/).

We first evaluated the performance of different variants of our algorithm and found that DEGAS usually identified the smallest pathways (**Text S1 and Figure S1**).

We next compared the results of DEGAS to those of three other methods for identifying pathways using network and expression data, jActiveModules [26], GiGA [31] and BioNet (implementing the algorithm described in [32]), and to t-test, which identifies sets of differentially expressed genes. jActiveModules and BioNet assign a differential expression score to every gene in every case, and then seek subnetworks with high aggregate scores [26]. jActiveModules selects a subset of samples for each module to maximize the score. GiGA first sorts all the nodes based on their differential expression score (e.g., the t-test p-value). Starting from the top ranked node, it iteratively adds the highest ranking node that is adjacent to at least one previously selected node. As GiGA requires the number of genes in the module to be set in advance, we set the size of GiGA modules to be the same as the best DEGAS module. When using t-test we selected either all the differentially expressed genes at FDR<0.05 (“t-test all”), or the same number of top differentially expressed genes as identified using DEGAS (“t-test top”). For each dataset we used each method to identify a module (or a set of genes for t-test) that is up-regulated, down-regulated or differentially expressed in the cases compared to the controls.

**Figure 3:**
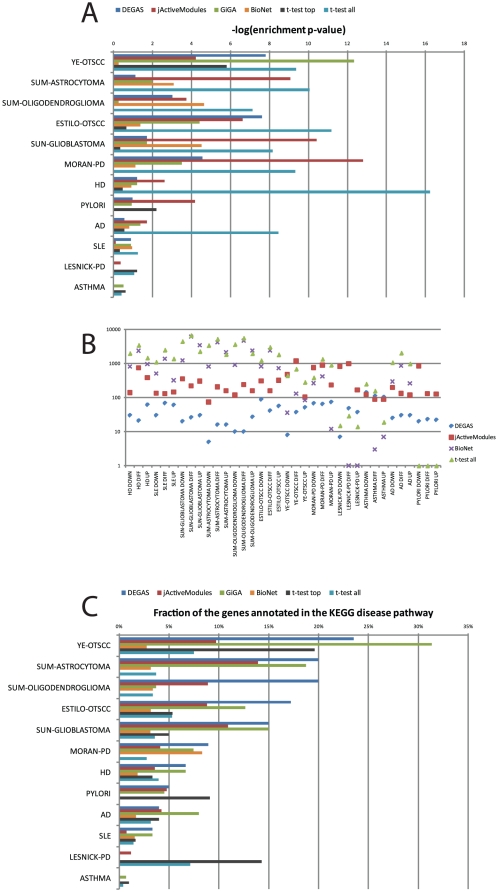
Comparison of methods for identifying disease-related pathways. For each dataset, each of the six methods was used to identify a module up-regulated, down-regulated or differentially expressed in cases compared to controls. The most significant module is shown for each method and each dataset, except those in which BioNet did not report any module. (A) The significance of the overlap between the obtained module and the KEGG disease pathway. (B) Comparison of the sizes of the modules. All modules are shown (C) Comparison of the fraction of the module genes that also appear in the relevant KEGG pathway. Only the most significant module is shown for each dataset and for each method.

We first compared the significance of the overlap between the obtained module and the KEGG pathway using the hypergeometric test. For each method, the most significant p-value obtained (inspecting up-regulation, down-regulation or differential expression) is shown in **Figure 3A**. For three datasets (SLE, LESNICK-PD and ASTHMA) we found that all the methods failed to identify a module that overlapped with the KEGG pathway (as we show later, in at least one of those cases (LESNICK-PD) we identify multiple potential biological insights from the DEGAS module). In the other datasets, jActiveModules performed best, outperforming the other methods in four cases. However, we found that the modules identified by jActiveModules were very large (**Figure 3B**), typically an order of magnitude larger than those of DEGAS, making them very difficult to interpret and use for derivation of biologically or clinically relevant insights. The hypergeometric test is known to be biased for larger modules, as they can give rise to much more significant overlaps. For example, in the MORAN_PD dataset, the most significant jActiveModules module contained 756 genes, only 31 of which (4%) were a part of the PD pathway in KEGG. The DEGAS module in this case contained only 67 genes, 9% of which were known to be PD-related. Indeed, we found that when comparing the fraction of the module that corresponded to the known KEGG pathway, DEGAS consistently outperformed jActiveModules, and performed better than all the competing methods in six datasets (**Figure 3C**). We thus conclude that DEGAS is capable of identifying small and focused modules that are more specific with respect to disease-related genes than those of jActiveModules, GiGA, BioNet or t-test.

### Pathway up-regulated in tongue carcinoma captures hallmarks of cancer

**Figure 4:**
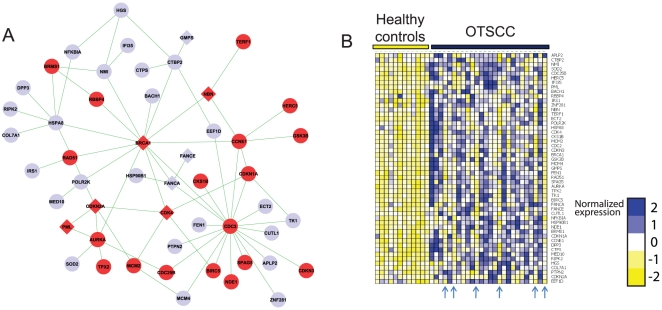
A dysregulated pathway in oral tongue squamous cell carcinoma. (A) The subnetwork of YE-OTSCC-UP, up-regulated in patients with oral tongue small cell carcinoma (OTSCC). Genes annotated with “cell cycle” in GO are in red. Diamond shaped nodes correspond to genes frequently mutated in cancer (taken from CGP [50]). (B) Expression patterns of the genes in the pathway. The expression pattern of each gene was normalized to mean 0 and standard deviation of 1. Arrows indicate six outlier samples selected by DEGAS.

We now focus on a 51-gene subnetwork identified by DEGAS as up-regulated in oral tongue squamous cell carcinoma (OTSCC), using a gene expression dataset due to Ye et al. [45] (YE-OTSCC-UP, k = 30 and p<0.005, **Figure 4**). As expected from a pathway up-regulated in quickly proliferating cells, this pathway is significantly enriched with genes annotated with “cell cycle” (p = 6.72·10^−14^) and “regulation of cell cycle” (p = 6.57·10^−8^) in GO. The most enriched KEGG annotations in this pathway are “Cell cycle” (p = 2.33 ·10^−9^) and “Pathways in cancer” (p = 1.6·10^−8^). In addition, it contains several members of key canonical oncogenic pathways (taken from MSigDB and KEGG), such as ATM (2.98·10^−8^), ATR/BRCA (p = 5.08·10^−8^) and p53 (1.25·10^−5^). Despite the fact that this pathway was discovered without using any genetic data, it was enriched with genes frequently mutated in cancer (taken from Cancer Gene Census [50], p = 1.34·10^−4^). Finally, the pathway was also enriched with genes whose disruption causes tumorgenesis in mice (taken from Mammalian Pheotype Ontology [51]). Taken together, these enrichments show how DEGAS can identify a focused subnetwork that contains the hallmarks of oncogenesis using a protein interaction network coupled with gene expression data comparing tumors to matching healthy tissues. Note also that several OTSCC samples show no evident dysregulation of the pathway, and they are automatically detected and excluded as outliers by DEGAS (**Figure 4**).

### Pathways dysregulated in Parkinson's disease

Parkinson's disease (PD) is the second most common progressive neurodegenerative brain disorder in humans, after Alzheimer's disease. PD has higher prevalence in males and affects 1 in 100 persons beyond 65 years of age. Pathologically, PD is characterized by degeneration of dopaminergic neurons in the *substantia nigra pars compacta* (SN), which leads to the depletion of dopamine in its striatal projections, which in turn leads to disruption of the cerebral neuronal systems responsible for motor functions [52]. This neurodegeneration is accompanied by the appearance of cytoplasmic inclusions called Lewy bodies in the surviving neurons in the SN as well as other regions of the central nervous system (CNS). The mechanism underlying the formation of these bodies and their pathological significance are largely unknown. Mutations in several genes have been linked to PD, but they explain less than 10% of the PD cases, and the mechanism of disease progression is still largely unknown [53].

**Figure 5:**
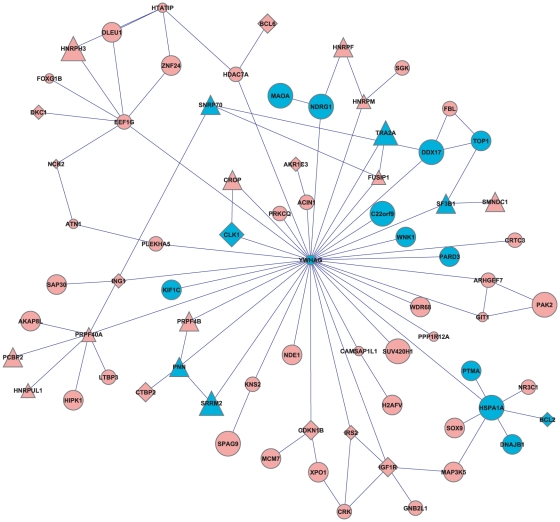
A DP of genes up-regulated in Parkinson's disease patients in the Moran et al. data. Nodes that appear also in the DP for k = 10 are in blue, the radius of each node is proportional to the number of patients in which it is dysregulated. Triangles are genes involved in mRNA splicing, diamonds are genes involved in cell proliferation.

We first focused on the PD expression dataset of Moran et al. [42], as it contained more samples than Lesnick et al. [43]. Using these expression profiles, we identified a 73-gene pathway as the most significantly up-regulated pathway in PD (MORAN-PD-UP, **Figure 5**). It was strikingly enriched with genes related to splicing– it contained 15 genes annotated with RNA splicing in GO “biological process” (p = 1.17·10^−10^, FDR<0.1). The module was identified for k = 30, but similar enrichments were seen in the pathways identified for k values between 25 and 10 (The core pathway dysregulated for k = 10 is highlighted in **Figure 5**). These results thus suggest a major up-regulation of the splicing machinery in PD. The literature contains several additional lines of evidence that splicing is affected in PD. Several studies found that the splicing of several of the key genes in PD, α-synuclein, parkin, synphilin-1, FOSB and RGS9, are affected in diseased individuals and in mouse models of the disease [54], [55], [56]. Furthermore, DJ-1, one of the genes mutated in genetic PD, has been implicated in splicing, through regulation of the splicing of tyrosine hydroxylase by the protein-associated splicing factor (PSF) [57]. Mitochondrial damage, a common phenomenon of several neurodegenerative diseases, including PD, Alzheimer's disease (AD) and Amyotrophic lateral sclerosis (ALS), was shown to affect alternative splicing in neural cells by increasing the relative abundance of shorter isoforms [58]. Finally, a recent study used three PD microarray datasets that were not used in our study [59], [60], [61] and identified the splicing factor SRRM2 as the only gene that was dysregulated in PD in all three datasets [62]. The latter study also identified hundreds of alternative splicing events in the blood of PD patients. However, we are not aware of any previous reports on a concerted up-regulation of parts of the splicing machinery in PD patients.

GO is a powerful tool for annotation of gene functions, but genes sharing a GO annotation are not always part of the same transcriptional program. In addition, GO does not contain information about human disease. In order to test if MORAN-PD-UP reflects such transcriptional programs, including those affected in diseased individuals, we utilized the curated gene sets from MSigDB [12] (**Table 2**). The most significant enrichment for an MSigDB term in MORAN-PD-UP was ALZHEIMER_DISEASE_UP (p = 1.57·10^−8^), which represents the set of genes up-regulated in the CA1 region of the hippocampus in individuals with AD [63]. This finding supports the notion that the pathways underlying neurodegenerative diseases, and in particular AD and PD, are similar [64]. The second most significant enrichment was for cell cycle and cell proliferation (PROLIFERATION_GENES, p = 2.84·10^−5^). The sub-module of proliferation genes in MORAN-PD-UP included several key cell cycle regulators such as p27 (CDKN1B), IGF1R, BCL2 and BCL6. At least four of these genes are known inhibitors of cell growth (CDKN1B, ING1, BCL6 and BCL2, annotated with “negative regulation of cell size” in GO). MORAN-PD-UP was also slightly enriched with genes involved in cell death (taken from GO, p = 0.001). The presence of these genes in MORAN-PD-UP indicate that over-expression of a network of genes involved in cell death could contribute to the loss of neurons that characterizes PD. Interestingly, this proliferation-related sub-module was almost entirely disjoint from the genes involved in RNA splicing, as only two genes, SMNDC1 and CROP, were shared between them (**Figure 5**). This may indicate that the splicing and the anti-proliferation modules are in fact separate. However, a recent study has implicated a splicing factor SRPK2 in neuronal cell death through regulation of cell cycle progression [65]. Interestingly, this regulation involves the 14-3-3 complex, a subunit of which, YWHAG, is the major hub in MORAN-PD-UP (see below). SPRK2 does not appear in MORAN-PD-UP, but the prominent presence of splicing-related and cell cycle-related genes, as well as a 14-3-3 component in this network, suggest that the role of up-regulation of splicing machinery, regulating cell cycle progression and leading to neuronal death, could be more important than previously appreciated.

**Table 2:** MSigDB terms from the “curated gene sets” collection that were enriched in MORAN-PD-UP.

MSigDB category	p-value
**ALZHEIMERS_DISEASE_UP**	1.57·10^−8^
**PROLIFERATION_GENES**	2.84·10^−5^
**SIG_PIP3_SIGNALING_IN_CARDIAC_MYOCTES**	9.02·10^−5^
**AGEING_BRAIN_UP**	1.78·10^−5^
**RCC_NL_UP**	4.19·10^−4^
**HADDAD_HSC_CD10_UP**	4.83·10^−4^
**UVC_HIGH_D8_DN**	4.92·10^−4^
**FLECHNER_KIDNEY_TRANSPLANT_REJECTION_DN**	5.26·10^−4^
**SHEPARD_NEG_REG_OF_CELL_PROLIFERATION**	5.90·10^−4^
**UVB_NHEK1_DN**	6.24·10^−4^
**HADDAD_HPCLYMPHO_ENRICHED**	6.59·10^−4^
**UVB_NHEK1_C6**	6.76·10^−4^
**PENG_GLUTAMINE_DN**	6.80·10^−4^

Only annotations with FDR<0.1 are shown.

**Figure 6:**
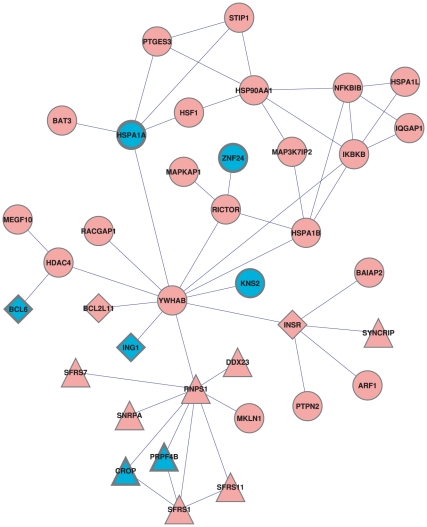
A DP of genes up-regulated in Parkinson's disease patients in the Lesnick et al. data. Nodes in common with MORAN-PD-UP are in blue. Triangles are genes involved in RNA splicing (taken from GO). Diamonds are genes involved in regulation of growth (taken from GO).

A recent study has found little overlap in the gene lists reported by different studies of the PD transcriptome [66]. In order to test the consistency of the results in another dataset, we analyzed another PD dataset due to Lesnick et al. [43], in which expression data from 16 PD cases were compared to 9 controls. The most significant subnetwork (LESNICK-PD-UP) was found for k = 25 (p<0.002) (**Figure 6**). Strikingly, LESNICK-PD-UP indicated the same enrichments (contained parts of the same pathways) as MORAN-PD-UP. It was significantly enriched with RNA splicing (1.42·10^−7^, FDR<0.1). Consistent with the anti-proliferation trend identified in MORAN-PD-UP, we also found a slight enrichment for “regulation of growth” genes in LESNICK-PD-UP (p = 0.006), with two genes known to be negative regulators of growth – ING1 and BCL6, shared with MORAN-PD-UP.

### 14-3-3 subunits are hubs in both PD up-regulated pathways

The main hubs in MORAN-PD-UP and LESNICK-PD-UP were YWHAB and YWHUG, respectively, the beta and the gamma polypeptides of the 3-monooxygenase 5-monooxygenase protein (14-3-3β and 14-3-3γ, respectively). In both networks, the 14-3-3 subunits were not significantly altered in most patients, but their neighborhoods were significantly dysregulated. We note that the neighborhoods of the two genes are overlapping (103 out of 169 nodes adjacent to YWHAB are also adjacent to YWHAG), and it is likely that both YWHAB and YWHAG take part in the same dysregulated pathway. Thus, the subnetworks we identified in both studies link 14-3-3 subunits to PD. Another 14-3-3 subunit, 14-3-3ξ, was shown to localize to Lewy bodies and to regulate parkin, a gene mutated in a subset of the genetic cases of PD [67], [68].

Lewy bodies, a hallmark of PD brain, contain aggregates of α-synuclein, which has a structural homology to 14-3-3 and binds it. Furthermore, the 14-3-3 subunit 14-3-3ξ was shown to localize to Lewy bodies [69], and 14-3-3 proteins were shown to bind proteins that are also bound by α-synuclein. This indicates that at least some of the network neighbors of 14-3-3 subunits that appear in the MORAN-PD-UP and LESNICK-PD-UP are also uncharacterized neighbors of α-synuclein. 14-3-3 also plays a role in regulation of dopamine biosynthesis through its regulation of tyrosine hydroxylase [70]. Previous studies have failed to identify genetic mutations in 14-3-3 subunits [69]. Our results indicate that the involvement of 14-3-3 in PD could be due to transcriptional dysregulation of the proteins it interacts with rather than due to protein integrity or expression levels.

### Evidence of stress response in PD up-regulated pathways

LESNICK-PD-UP contained three Hsp70 proteins HSPA1A, HSPA1B and HSPA1L, as well as additional stress-related genes HSF1, STIP1, PTGES3 and HSP90AA1. This network was also enriched for predicted targets of the Hsf1 transcription factor (p = 1.1·10^−5^, FDR<0.1, taken from MSigDB). HSP1A also appeared in MORAN-PD-UP, which was also enriched with predicted Hsf1 targets (p = 1.5·10^−3^, FDR<0.1). Up-regulation of Hsp-related proteins has also been noted in another study of the PD *substantia nigra* transcriptome, the data of which was unfortunately not available [71]. This up-regulation of the heat-shock response, observed in multiple studies, is consistent with the hypothesis that this response is activated as a result of the aberrant protein folding occurring in PD.

### Pathway down-regulated in PD contains hallmarks of the disease

We also identified significantly down-regulated pathways in both PD datasets. Since the most significant pathway identified in the Lesnick et al. study was very small (7 genes), we will focus here on the pathway identified in the Moran et al. dataset. (p<0.002), which was identified for k = 30 and contained 67 genes (**Figure S2**). This pathway contained several hallmarks of PD. It was enriched with genes from the KEGG PD pathway (p = 2.72·10^−5^, FDR<0.1), as well as with genes annotated with “Parkinson's disease” in Entrez Gene (including GeneRIFs [72], p = 3.58·10^−4^). In contrast, the up-regulated PD was not enriched with either of those PD-related gene sets, perhaps as it contains a novel biological finding. Neither the up-regulated nor the down-regulated PDs were enriched with genes mutated in genetic PD (taken from OMIM [73]). Consistent with our findings in MORAN-PD-UP, we found that the MSigDB curated gene set most significantly enriched in MORAN-PD-UP contained genes down-regulated in Alzheimer's disease (ALZHEIMERS_DISEASE_DN, p = 2.28·10^−12^). Huntington's disease, another neurodegenerative disease was highly represented in MORAN-PD-DOWN. The major hub in this pathway is huntingtin (HD), mutations in which cause this disease. In addition the KEGG Huntington's disease pathway was significantly enriched in MORAN-PD-DOWN (p = 3.32·10^−6^). GO enrichment analysis also pointed towards common neural functions such as learning (p = 3.86·10^−8^) and synaptic transmission (p = 4.56·10^−7^). These suggest that one of the reasons for the down-regulation of at least some of the genes in MORAN-PD-DOWN could be loss of neuronal cells, which is known to confound transcriptome studies of the SN in PD patients [66]. The second major theme in MORAN-PD-DOWN was oxidative phosphorylation, with five genes involved in this process (CYC1, UQCRC2, NDUFS1, NDUFA9, NDUFV2), all of which also appear in the KEGG PD pathway. Down-regulation of these genes is a well characterized feature of PD [71].

## Discussion

We developed a novel computational technique for network-based analysis of case-control gene expression data. The method is aimed at identifying pathways in the interaction network that exhibit ample evidence of disruption of transcription that is specific to diseased patients, but without requiring that any gene is significantly differentially expressed across all the cases. Application of the method to a large-scale protein-protein interaction network and expression data from seven human diseases has shown its potential in outlining subnetworks with a high relevance to the mechanisms of pathogenesis. Comparison to extant techniques for analysis of gene expression data highlights the advantages of our approach in identifying clinically sound pathways.

While the results presented here are encouraging, there is certainly room for further development of these methods, which can be extended in several directions. First, we currently report only a single subnetwork for each pathway, whereas clearly, in some diseases, multiple distinct pathways can be affected. One possible way of seeking multiple subnetworks is to iteratively find and remove the most significant DP from the network. Better methods are needed to detect overlapping DPs. One possible alternative is to start the search procedure from multiple starting points simultaneously, thus “growing” in parallel several DPs.

Another fundamental difficulty in identifying protein interaction subnetworks using expression data is inclusion of genes that are not significantly affected on the expression level, but are required for subnetwork connectivity. We have previously coined the term “back nodes” for such nodes (as opposed to “front nodes”, whose expression level shows variation across the profiles) [23]. Since in most datasets, only a minority of the genes show significant expression changes, usually there is a large number of possible back nodes, and choosing the most relevant ones poses a difficult challenge. This challenge is made more difficult by the scale-free nature of protein interaction networks, which contain a small number of hubs with large degrees [74]. These hubs have a much higher tendency to be included as back nodes. In DEGAS, we attempt to address this problem by removing from the networks hubs that are not relevant for the studied dataset (see Methods). We found that this approach helps to avoid adding irrelevant back nodes, while still allowing highly connected proteins to appear in DPs. We believe there is room for further improvement of this approach, in order to include only disease-related hubs in the dysregulated pathways.

Finally, our problem formulation used a fixed *k* value, thus requiring that the same lower bound on the genes altered in each patient. All the algorithms and proofs presented here and in the **Text S1** are generalizable to the scenario where different samples have different thresholds, but specifying such thresholds remains a difficult problem open for further investigation.

One of our main goals was to develop a method that will allow *de novo* detection of pathways affected by human disease, without requiring that individual genes in the pathway are differentially expressed. This approach is motivated by several recent studies that have shown that human diseases have relatively few genes that are frequently affected in cases, but that mutations tend to cluster in specific disease-related pathways [7], [8], [9], [10]. Here we use gene expression to define gene dysregulation. Naturally, our approach can be extended to other definitions of dysregulation, in particular genetic dysregulation by SNPs and copy number changes, which are now extensively studied on a genome-wide scale. The challenge in this extension is the distinction between mutations that disrupt the activity of the gene and “passenger” mutations that have little effect. Furthermore, it is highly desirable to develop a method that will be able to detect subnetworks affected at different levels, including genetic alternations, transcription and post-transcriptional and post-translational regulation. Measuring some of the relevant quantities (e. g. protein expression) on a genome-wide scale will require advancement of experimental methods beyond what is possible today.

Our analysis of gene expression in the substantia nigra of PD patients highlights the significant up-regulation of splicing machinery and negative regulators of cell proliferation. Importantly, the results we describe here could not be obtained using a standard statistical approach. At FDR<0.05, 34 genes are found as up-regulated in the Lesnick et al. study showing no significant enrichment for RNA splicing (0.046 before correction for multiple testing). 377 genes are found as up-regulated in the Moran et al. study and they are significantly enriched for RNA processing, but show no significant enrichment for cell proliferation. The two sets obtained using a t-test on both datasets have only 13 genes in common. The overlap between the two DEGAS pathways up-regulated in PD in those two datasets was 12.7%, compared to just 5.5% for t-test.

We believe that we have presented here a novel and important approach for using PPI networks in human case-control gene expression studies. Numerous confounding factors can make the discovery of robust disease signatures difficult. Our use of a PPI network places the dysregulation of each gene in the context of the dysregulation of its neighbors and allows detection of a pathway dysregulation signature, which is more robust and more biologically relevant. As the quality and the extent of both gene expression datasets and, more importantly, the human PPI network are expected to vastly improve, we believe that this approach will be widely adopted.

## Methods

### Basic graph theoretic definitions

We first define several basic graph theoretic terms. Unless indicated otherwise, all the terms refer to the input graph *G* = (*V*,*E*), which represents the protein interaction network. *N(v)* is a set of neighbors of *v* in G. Given two nodes *v* and *u*, the *distance* between *u* and *v,* denoted d(*u*,*v*), is the length in edges of a shortest path between *u* and *v* in the graph. The *r-neighborhood* of a node *v* is the set of all the nodes in the graph at distance ≤*r* from *v*. The *radius* of a graph is the least r such there exists a node whose r-neighborhood contains the entire graph. Equivalently, it is the value min_v∈V_max_u∈V_{*d*(*v*,*u*)}. A Breadth First Search (BFS), is a graph traversal algorithm that starts at a node *v_1_* in the graph and iteratively scans the graph such that in iteration *i* it visits the nodes that are at distance *i* from *v_1_*. A *BFS tree* is a graph in which each node is connected to the node in the previous level of the BFS search that was used to discover it. See [75] for more details.

### The Connected Set Cover problem

We formalize the problem of finding DPs as follows. We are given an undirected graph *G* = (*V*,*E*) and a collection of sets {S_v_}_v∈V_ over the universe of elements *U*, with |*U*| = *n*. In our biological context, *U* is the set of the cases, and *S_v_* is the set of cases in which gene *v* is dysregulated. For ease of representation, we will use, in addition to *G*, a bipartite graph *B* = (*V*,*U*,*E^B^*) where for v∈V, u∈U (*v*,*u*)∈E^B^ if and only if u∈S_v_ (**Figure 1D**). A set C⊆V is a *connected (k,l)-cover* (denoted *CC*(*k*,*l*)) if *C* induces a connected component in *G* and a subset *U'*⊆*U* exists such that |*U'*| = *n*-*l* and for all *u'*∈*U'*, |*N*(*u'*) ∩*C*|≥*k*, i.e., in the induced subgraph (*C*,*U'*) (the subgraph in B that contains all the edges of E^B^ between the node sets C and U') the minimal degree of nodes in *U'* is at least *k*. We are interested in finding a *CC*(*k*,*l*) of the smallest cardinality. We denote this minimization problem by *MCC*(*k*,*l*).

### Similar problems

Given a universe W of n elements and a collection of sets S_1_,...,S_m_⊆W, the *set cover problem* is to identify a smallest collection of sets such that all the elements are included in their union. If G is a clique (fully connected), every C⊆V is connected, and therefore *MCC*(1,0) is equivalent to the set cover problem. For this classical NP-hard problem, Johnson proposed a simple greedy algorithm with approximation ratio *O*(ln(*n*)) [76]. This ratio is the best possible unless P has slightly super-polynomial time algorithms [77]. If *k*>1 and *G* is a clique, the *MCC*(*k*,0) problem is equivalent to the *set multicover problem*, also known as the set *k*-cover problem, a variant of the set cover problem in which every element has to be covered *k* times. The set multicover problem can be approximated to factor of *O*(*p*), where *p* is the number of sets covering the element that appears in the largest number of sets [78]. The greedy algorithm for set multicover was shown to achieve an approximation ratio of *O*(log(*n*)) [79]. See [78] for a comprehensive review of the available approximation results on set cover and set multicover problems.

For a general *G*, *MCC*(1,0) is the *connected set cover problem*, which has been recently studied in the context of wavelength assignment of broadcast connections in optical networks [80]. It was shown to be NP-Hard even if at most one vertex of *G* has degree greater than two, and approximation algorithms were suggested for the cases where *G* is a line graph or a spider graph. Neither of these special cases is applicable in our biological context.

### The Minimal Radius Connected Set Cover problem

An initial analysis using the basic formulation has produced results that are not always satisfactory from the biological standpoint (results not shown). We also found that biologically relevant subgraphs generally tend to have small average shortest path. As minimizing or constraining the average shortest path of a graph is difficult, we chose to look for a minimal set that also had a small radius. In this study we thus aim to solve the *minimal radius minimal connected set cover* (*MRMCC*) problem, which is the following problem: Let r_min_ be the minimum value such that there exists S⊆V that is a CC(k,l) of radius r_min_. Then MRMCC seeks a minimum cardinality CC(k,l) of radius r_min_.

### MRMCC(k,l) is equivalent to MCC(k,l) in terms of computational complexity

We now show that an algorithm solving *MCC*(*k*,*l*) can be efficiently used to solve *MRMCC*(*k*,*l*). If there exists a CC(k,l) of radius r_min_, it contains a node whose r_min_-neighborhood contains a CC(k,l). Our method will focus on finding such nodes. r_min_ can be efficiently found in polynomial time using the following procedure. We initialize an empty array *A* with *n* entries and starting from every possible root node *v*, use BFS on G to find all the nodes at distance *i* from *v* for *i* = 1,2,3,…. When each node is reached, the entries in *A* corresponding to the elements it covers are incremented. After all the nodes in a level of BFS have been scanned, use *A* to check if at least *n-l* elements have been covered at least *k* times. This condition is met for the first time when *i* equals the smallest *r* for which a CC(k,l) is found in the r-neighborhood of v. After this procedure is executed for each v∈*V*, we can identify the value of r_min_, and the set V_min_ of nodes that contain a *CC*(*k*,*l*) in their *r_min_*-neighborhood. Clearly, the optimal solution to *MRMCC*(k,l) must be contained within the *r_min_*-neighborhood of one of the nodes in *V_min_*, and if we solve *MCC(k,l)* for each of those r_min_-neighborhoods, we will obtain an optimal solution to *MRMCC*(*k*,*l*). We can thus identify *r_min_* and *V_min_* using |*V*| executions of the BFS algorithm, each taking *O*(|*V*|+|*E*|). *MRMCC*(*k*,*l*) can thus be reduced to *MCC*(*k*,*l*) in polynomial time. Practically, we use the approach described above to solve *MRMCC*(*k*,*l*) (**Figure 2**). To speed up the search for *V_min_*, after a subset of the nodes has been tested as potential roots, if the currently smallest radius is r_min_*, for all subsequent root nodes, we halt the BFS procedure when it reaches level r_min_*+1.

### ExpandingGreedy algorithm for MCC(k,l)

We now describe a heuristic called ExpandingGreedy for solving MCC(k,l), which is used in DEGAS. This was one of several algorithms we developed for the problem and it proved best in practice. The other algorithms for MCC(k,l) and their comparison are described in the **Text S1**. ExpandingGreedy is motivated by the greedy approximation algorithm for the set cover problem [77]. It works as follows: Given a partial cover *W*⊆*V* and the set of corresponding k-covered elements *X*⊆*U*, the algorithm picks a node *v*∈*V* that is adjacent to *W* and that covers the largest number of elements of *U*\*X* and adds *v* to the cover. In case of a tie, the nodes are ranked based on the total number of elements covered by their neighbors, and the best node is selected. Initially *W* = Ø, *X* = Ø and the first node is picked without connectivity constraints.

Unfortunately, *ExpandingGreedy* can be shown in some instances to give a solution that is O(|V|) times the optimal solution for MCC(1,0) (**Text S1, Figure S3**). However, augmented with powerful heuristics, some of which are described below, our extensive testing shows that it performs better than other algorithms for MCC(k,l) (**Text S1 and Figure S1**).

### Practical heuristics and implementation details

In order to improve the performance of DEGAS, we implemented several practical heuristics, which significantly decrease the size of the obtained DPs (see **Text S1**).

- ExpandingGreedy*2. Since ExpandingGreedy always selects the node covering the most uncovered elements, the set of *l* outliers is not selected until the algorithm halts. Because of this, ExpandingGreedy may add superfluous nodes covering elements that will eventually be discarded as outliers. This problem can be partially addressed by choosing a set of “good” outliers in advance. We therefore used the following heuristic – we first ran the ExpandingGreedy algorithm and identified the set of *l* outliers *O*⊆*U*. We then ran the same algorithm hiding the nodes in *O*, and setting *l* to zero. This heuristic sometimes significantly reduced the number of nodes in the cover (**Figure S1**).
- **Hub hiding.** One of the key challenges for methods based on connectivity in PPI networks in mammals is the biased nature of the known networks, in which heavily studied genes, such as p53, are highly connected “hubs” in the network. In some cases this high connectivity has biological meaning, but in others, it could merely be the result of more extensive testing of interactions for some genes. This issue requires special attention, as a simplistic algorithm will include those hubs in the solution, even if they are not related to the studied disease. In order to avoid irrelevant yet highly connected nodes, we introduced a preliminary step in which hubs were removed from the network, unless the node and genes in its direct neighborhood (i.e., the network nodes adjacent to the hub) experienced many dysregulation events. Specifically, we filtered out nodes with degree >100, for which the average number of covered elements in their direct neighborhood was not in the top 25%, compared to all the direct neighborhoods in the graph.
- **Clean-up heuristic.** The DPs produced by ExpandingGreedy may contain superfluous nodes that are necessary neither for the cover requirements nor for subnetwork connectivity. We therefore perform a clean-up step that iteratively removes such nodes, while maintaining network connectivity, until no further reduction is possible. This step is applied also to all other algorithms described in the **Text S1.**

### Parameter setting

To select the *k* value, 200 random networks were generated by random shuffling of the gene names of the nodes in G. DEGAS was then executed on each network, for a range of values of *k*, and an empirical p-value was computed as the fraction of these 200 networks in which DEGAS found a smaller DP than the one found in the real network. The *k* for which the size of the DP was most significant was subsequently used. In case of a tie, a normal distribution was fitted to the random scores, and k yielding the subnetwork with the most significant *z*-score was selected.

### Gene expression data and specification of dysregulated genes

All gene expression datasets were obtained from GEO [1]. The original normalization of each dataset was used, and values were log-transformed if necessary. Probes corresponding to the same EntrezGene identified were averaged, and genes which did not appear in the network were discarded. For each gene, the average and the standard deviation of its expression in the control samples were computed. These were used to fit a normal distribution and to compute a p-value for the expression of the gene in each case sample. The gene was considered differentially expressed if this p-value was <0.05 and the ratio between its expression level in the case sample and the average expression in the controls was at least 1.4.

### Human protein interaction network

We compiled a human protein-protein interaction network encompassing 10,682 nodes corresponding to Entrez Gene identifiers and 50,185 interactions. The interactions are based mostly on small-scale experiments and were obtained from several interaction databases. The network is available at the supplementary website http://acgt.cs.tau.ac.il/degas. A list of sources used to create the network and the number of interactions from each source in the network appears in **File S1**.

### Implementation of other methods

jActiveModules was applied using Cytoscape [81] to the p-values computed for each case by fitting a normal distribution to the gene expression levels in the controls. The top scoring module was selected for further analysis. GiGA was implemented as described in the original manuscript [31], and the module size was set to equal the module size identified by DEGAS. The method of Dittrich et al. [32] was applied using its implementation in the BioNet R package. The *runFastHeinz* heuristic was used with the FDR set to 0.01.

### Implementation details

A JAVA implementation of DEGAS is integrated into the MATISSE software package alongside implementations of other algorithms combining network and gene expression data [22], [23], [82]. This implementation allows the user to set all the parameters described in this paper, to execute the different algorithms described here and in the **Text S1** and dynamically view the resulting dysregulated pathways.

## Supporting Information

Text S1 Supplementary Methods
(0.06 MB DOC)

Figure S1 Comparison of cover sizes found by different algorithms for MCC(k,l). Each dataset is represented by three rows corresponding to identifying DPs up-regulated, down-regulated or differentially expressed. For each dataset and each dysregulation direction, we ranked the cover sizes obtained by each algorithm according to their size. The smallest cover was assigned rank 0, and the largest rank 1. (B) The averages of the ranks shown in (A) for each algorithm.
(2.71 MB EPS)

Figure S2 A DP of genes down-regulated in Parkinson's disease patients in the Moran et al. data. Nodes annotated with transmission of nerve pulse in GO are in blue. Triangles are genes that appear in the Parkinson's disease pathway in KEGG.
(1039 KB EPS)

Figure S3 A worst case scenario for the performance of ExpandingGreedy for MCC(1,0).
(0.07 MB TIF)

File S1 Sources of interactions in the protein-protein interaction network
(1.99 MB XLS)
