# Supplementary material for: DEGAS: De Novo Discovery of Dysregulated Pathways in Human Diseases
Source: PLoS One. 2010 Oct 19;5(10):e13367. doi: 10.1371/journal.pone.0013367 (PMC2957424; doi:10.1371/journal.pone.0013367)
Supplement: Text S1 — Supplementary Methods (0.06 MB DOC) [file pone.0013367.s001.doc]

**Supplementary Text**

**Worst case performance of ExpandingGreedy**

Recall that *ExpandingGreedy* maintains a partial cover *W*⊆*V* and the set of currently k-covered elements *X*⊆*U*. Initially *W*= ∅, *X*=∅. In each iteration, the algorithm picks a node *v*∈*V* that is adjacent to *W* and that covers the largest number of elements of *U*\*X* and adds *v* to the cover. By picking v adjacent to W, the connectivity of the cover is preserved.

*ExpandingGreedy* can give a solution that is Θ(|V|) times the optimum for MCC(k,l): Consider the example in **Supplementary Figure 3**. The smallest cover is {X,Y_1_,Z}. The algorithm will start from X as it provides the best initial cover of the elements. However, afterwards, in the worst case, the algorithm adds to the cover Y_2_,...,Y_n_ before Y_1_ and finally Z are added. In this case the obtained cover size will be |V|, i.e., Θ (|V|) times the optimum.

In order to handle such scenarios more successfully, we implemented a look-ahead heuristic: in case of such ties, the neighborhood of the candidate nodes is explored and the node whose neighborhood provides the best coverage of uncovered elements is selected.

**Other algorithms for solving MCC(k,l)**

In addition to ExpandingGreedy, we developed several additional algorithms for the minimum connected set cover problem. These algorithms are described below.

**ConnectingGreedy**

We describe the algorithm for MCC(1,0) only. *ConnectingGreedy* initially ignores the connectivity constraints and uses the simple greedy algorithm [1] to find a small set cover X⊆V. It then augments this cover X with additional nodes in order to obtain a connected set cover*,* as follows: Initially Y=X. Let *C* be any connected component in the subgraph induced by Y. The algorithm randomly picks a node v∈Y\C and adds to Y the nodes along a shortest path from Y to v (including v). This is repeated until Y is connected. When the algorithm halts Y is a proper CC(1,0): (a) it is a proper cover as it includes X; (b) it induces a connected component in G.

Recall that the *diameter* of G is the maximum length of a shortest path between a pair of nodes in V.

*Claim:* *ConnectingGreedy* guarantees an approximation ratio of *O*(*D*log(*n*)) for *MCC*(1,0), where *D* is the diameter of *G*.

*Proof:* Let C_opt_ be an optimal solution to MCC(1,0) and let C* be an optimal solution to the regular (not connected) set cover problem. Clearly, |C_opt_|≥|C*|. Let X be the set of nodes obtained by the first phase of ConnectingGreey and let C_CG_ be the final solution when it terminates. Then |C_CG_|≤D|X| since connecting each two components in X requires at most D nodes. By the greedy approximation to the regular set cover problem it follows that |X|≤(ln(n)+1)|C*| and therefore |X|≤(ln(n)+1)|C_opt_|. In conclusion |C_CG_|≤D|X|≤D (ln(n)+1)|C_opt_|. ■

**The CUSP algorithm for MCC(1,0)**

We next describe an algorithm called Covering Using Shortest Paths (*CUSP*). We first give the algorithm for *MCC*(1,0) and then extend it for the general case. Roughly speaking, starting from an arbitrary node, the algorithm connects to it close nodes that cover different elements. For each node *r* the algorithm proceeds as follows. Denote by d(u,v) the shortest distance between nodes u and v in G(V,E). For every *u*∈*U*, it finds *P*[*r*,*u*], a closest node to *r* that covers *u*: *P*[*r*,*u*]=argmin*_w_*{d(*r*,*w*)|(*w*,*u*)∈ *E^B^*}. Let us denote by M[*r*,*u*] the distance from *r* to this node. The closest such nodes for all *u*∈*U* can be found in *O*(|*V*|+|*E*|+|*E^B^*|) by building a BFS tree *T* rooted at *r* [2]. Now define *X_r_* as the union of the paths in *T* from *r* to *P*[*r*,*u*] for every *u*∈*U*. *X_r_* is a proper *CC*(1,0): (a) it is a subtree of *T* and thus induces a connected component in *G*; (b) every *u*∈*U* is covered by P[r,u]. The final solution is obtained by choosing the minimum cardinality X_r_ : X=argmin_v_{|X_v_|}.

*Claim*: CUSP gives an n-approximation for MCC(1,0).

*Proof*: Consider an optimal solution C_opt_ and the solution C_cusp_ given by CUSP. Pick any node z∈C_opt_. As C_opt_ is a proper cover, for every u∈U, it must contain at least one node w_u_ for which (w_u_,u)∈E^B^. Let w_u_* be such a node for which d(z,w_u_*) is maximal. As C_opt_ induces a connected component in G and contains both z and w_u_*, it must also contain all the nodes on some path between z and w_u_*, and thus |C_opt_|≥d(z,w_u_*)+1=max_{u∈U}_{d(z,w_u_)|(w_u_,u)∈E^B^}+1 ≥ max_{u∈U}_{M[z,u]}+1

Recall that C_cusp_ is the minimum cover among all X_r_, and therefore |C_cusp_|≤|X_z_|. X_z_ is a union of shortest paths from nodes for which M[z,u] is minimal. The number of nodes on every such path is not longer than max_u∈U_{M[z,u]}+1 and the number of paths is n. Hence, |C_cusp_|≤n⋅(max_{u∈U}_{M[z,u]} + 1). Joining the two bounds together we get |C_cusp_|/|C_opt_|≤n. ■

Note that n=|U| can be substantially smaller than |V|, as is the case, for example, in our biomedical application. In terms of computational complexity, each iteration of CUSP requires an execution of BFS, assigning to each node in V its level in the tree, and for each *u*∈*U* its neighbor with the least level is found in *O*(|*N*(*u*)|). Hence, the total amount of work for each choice of r is *O*(|*V*|+|*E*|+|*E^B^*|) and the overall complexity is O(|*V*|(|*V*|+|*E*|+|*E^B^*|)). Note that it is not necessary to execute the algorithm from every root node: If *x*∈*U* is an element covered by the least number of nodes, it suffices to run CUSP only from nodes that cover *x*.

**The CUSP algorithm for *MCC*(*k*,*l*)**

The algorithm described above can be generalized for *k*>1, as follows. Instead of finding a single node closest to *r* that covers the element *u*, the algorithm will compute distances (M[*r*,*u*]_1_,...,M[*r*,*u*]*_k_*) and pointers (P[*r*,*u*]_1_,...,P[*r*,*u*]*_k_*) to the *k* closest such nodes. This can be done by computing the distances from *r* to all the nodes in *V*, and then retrieving the *k* closest nodes, which can be solved, e.g., in O(k|V|) by repeated minimum finding or in O(|V|log|V|) by sorting. The cover is again obtained by uniting all the paths to all these nodes. This algorithm is readily generalized for *MCC*(*k*,*l*) where l>0. The only change required is that only paths to nodes covering the *n*-*l* elements for which max*_q_*d(*r*,P[*r*,*u*]*_q_*), 1≤*q*≤*k* are the smallest enter the cover. Here too, it is not necessary to execute the algorithm from each *r*∈*V*, but only from the *l*+1 nodes that cover elements from *U'*⊆*U* for which max*_u'_*_∈_*_U'_*|*N*(*u'*)| is minimal.

*Claim*: the extended *CUSP* algorithm gives an k(n-l)-approximation for MCC(k,l).

*Proof.* Consider an optimal solution C_opt_ and the solution C_cusp_ given by CUSP. Let U_opt_, U_cusp_⊆U be the sets of elements covered by C_opt_ and C_cusp_ respectively (|U_opt_|=|U_cusp_|=n-l). Let D_i_[w,u] be the i-th smallest value in {d(w,v)|v∈V, (v,u)∈E^B^}. Let z be any node in C_opt_.

We first show that |C_opt_|≥ max_u∈Uopt_{D_k_[z,u]+1}. As C_opt_ is a proper cover, it must contain at least k nodes that cover each u∈U_opt_. Let us denote the set of these nodes V_u_. Let w_u_*∈∪_u_V_u_ be a node for which d(z,w_u_*) is maximal. As C_opt_ induces a connected component in G and contains both z and w_u_*, it must also contain all the nodes on some path between z and w_u_*, and thus: |C_opt_|≥d(z,w_u_*)+1=max_{u∈Uopt}_max_{v∈Vu}_{d(z,v)}+1≥max_u∈Uopt_{D_k_[z,u]}+1.

We now add an upper bound on |C_cusp_|: |C_cusp_| ≤ k(n-l) (max_{u∈Uopt}_{D_k_[z,u]}+1). C_cusp_ is the minimum cover over all X_r_, and therefore |C_cusp_|≤X_{z}_. X_{z}_ is a union of shortest paths to nodes covering n-l elements for which D_k_[z,u] was minimal and therefore max_{u∈Ucusp}_{D_k_[z,u]}≤max_{u∈Uopt}_{D_k_[z,u]}. The number of nodes on every shortest path is thus not more than max_{u∈Uopt}_{D_k_[z,u]}+1 and the number of paths is k(n-l).

Joining the two bounds together we obtain

|C_cusp_| ≤ k(n-l) [max_{u∈Ucusp}_{D_k_[z,u]} +1]≤ k(n-l) [max_{u∈Uopt}_{D_k_[z,u]}+1] ≤ k(n-l) |C_opt_|

Hence, the CUSP algorithm achieves an approximation ratio of k(n-l). ■

**The CUSP* heuristic**

We now describe a heuristic based on CUSP, called CUSP*. For each root r, it starts from a specific partial cover called a *core* - a set of μ nodes that can be computed efficiently and has good coverage properties, and then completes the cover as CUSP does.

Let the *total coverage* of a subset V'⊆V be the total number of edges between V' and U in B. Given a BFS tree T from r, we define a μ*-core* as a connected subtree T'=(V',E') rooted at r such that |V'|=μ and the total coverage of V' is maximal. An optimal μ-core can be found efficiently using dynamic programming, as follows. Let C[v,i] be the maximal total coverage of a connected subtree of T containing i nodes and rooted at v. C[v,i] is computed for each v∈V,1≤i≤μ as follows. We first set C[v,1]=|N(v)| and C[v,i]=0 for i>1 for every leaf v of T. Then, if v has p children v'_1_,...,v'_p_ in T and 1≤i≤μ: C[v,i]=|N(v)|+max_{(i1,...,ip)}_{Σ_1≤j≤p_C[v_j_,i_j_]|Σ_j_i_j_=i-1. The computation of all C[v,i] values is done by a post-order traversal of T. The optimal value is C[r, μ] and the corresponding subtree is reconstructed by maintaining the optimizing subsets for each v and i. The problem of computing max_{(i1,...,ip)}_{Σ_1≤j≤p_C[v_j_,i_j_]|Σ_j_i_j_=i-1} is an instance of the knapsack problem and can be computed in O(μp) for each v [3]. The overall complexity of finding a μ-core is thus O(μ^2^|V|).

For every root node r, CUSP* computes C[r,μ] and obtains the corresponding μ-core C_core_ using traceback. The set C_core_ is contracted in G into a single node x and the edges between x and U are removed from B. We then compute R=(R_1_,...,R_n_) where R_i_ = max{ 0 , k - |{(v,u)∈E^B^|v∈C_core_}|}. R_1_,...,R_n_ are the residual coverage requirements of the elements. The rest of the cover is obtained using CUSP. The best cover over all root choices is the solution.

Note that the notion of total coverage deliberately ignores the specific coverage of each node in U by the core and does not consider possible outliers, in order to be able to compute the core efficiently. Hence it should be applied with μ≤k, to allow the CUSP algorithm to complete the cover more carefully. In our implementation we first compute a k-core. Let *c_m_* be the maximum coverage of any element by the nodes in that core. If *c_m_<k*, we then extend this core by computing a (k-*c_m_*)-core on the contracted graph. We repeat the extension until at least one element is covered k times. We then proceed with applying the CUSP algorithm to complete the residual cover.

**Evaluating the performance the algorithms**

We applied the different algorithms that we developed for MCC(k,l) to each of the 13 datasets described in the main text and compared the sizes of the obtained covers. As each dataset is expected to give rise to covers of different sizes, for each dataset we ranked the size of the covers found by all algorithms. Mid-ranking were used in case of ties. We ran each algorithm with k=5, 10, 15, …, 40, ranked the solutions for each k according to the cover size and used the average rank as a goodness of fit measure. As shown in **Supplementary Figure 1**, the smallest covers were obtained when using the ExpandingGreedy algorithm, with further improvement when using the ExtendingGreedy*2 heuristic. Thus, despite the fact that this algorithm has a poor worst case performance, on biological data it performs best than algorithms with provable theoretical performance

1. Johnson D. Approximation algorithms for combinatorial problems; 1973. ACM New York, NY, USA. pp. 38-49.

2. Cormen TH (2009) Introduction to algorithms. Cambridge, Mass.: MIT Press. xix, 1292 p. p.

3. Lawler EL (1976) Combinatorial optimization : networks and matroids. New York: Holt, Rinehart and Winston. x, 374 p. p.
