# Supplementary figures and images for: DEGAS: De Novo Discovery of Dysregulated Pathways in Human Diseases
Source: PLoS One. 2010 Oct 19;5(10):e13367. doi: 10.1371/journal.pone.0013367 (PMC2957424; doi:10.1371/journal.pone.0013367)

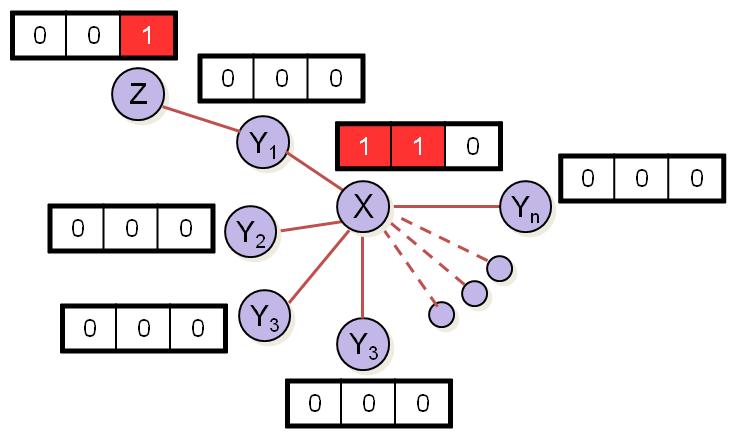

Supplement: Figure S3 — A worst case scenario for the performance of ExpandingGreedy for MCC(1,0). (0.07 MB TIF) [file pone.0013367.s004.tif]
